# Enhancing Self-Management Support Apps for Spinal Cord Injury: The Missing Role of Caregivers

**DOI:** 10.2196/72037

**Published:** 2025-04-14

**Authors:** Cheila Pessoa

**Affiliations:** 1 Spinal Cord Injury Rehabilitation Unit, Centro de Reabilitação do Norte - Dr. Ferreira Alves (CRN), Valadares, Centro Hospitalar de Vila Nova de Gaia, Avenida Infante Sagres 349, Vila Nova de Gaia, 4405-565, Portugal, 351 930417512; 2 Escola Superior de Enfermagem do Porto, Porto, Portugal

**Keywords:** mobile phone, mHealth, eHealth, telemedicine, telehealth, spinal cord injury, self-management, internet-based intervention, World Wide Web, systematic review, caregiver

I read, with great interest, the recent article by the team from Swiss Paraplegic Research, Nottwil, Switzerland, titled *Self-Management Support Apps for Spinal Cord Injury: Results of a Systematic Search in App Stores and Mobile App Rating Scale Evaluation* [1].

The authors conducted a comprehensive review of available mobile apps aimed at supporting self-management for individuals with spinal cord injury (SCI). Their work provides valuable insights into the current landscape of digital tools in this domain.

As a rehabilitation nurse specializing in SCI at the North Rehabilitation Centre of Portugal, having worked in this field since 2017, I have dedicated my career to researching the rehabilitation process of patients with SCI, particularly the training and empowerment of caregivers [2]. This experience, coupled with my research and app development background, informs my perspective on the study by Bernard et al [1].

I commend the authors for their rigorous systematic search of mobile apps designed to support self-management for individuals with SCI. However, I would like to highlight a critical aspect that may warrant further consideration: the exclusion of caregiver-centered apps. Many patients with SCI, particularly those with cervical injuries, experience severe functional limitations that hinder their ability to independently use self-management apps. As emphasized by Armstrong-Wood et al [3], individuals with high-level SCI may struggle with smartphone accessibility, which underscores the importance of caregiver involvement in the creation of these apps and caregivers’ presence during app usage.

A crucial point raised in the study is the role of technological companies in developing self-management apps without direct involvement from SCI specialists or end users. While collaborations with researchers are mentioned, it is essential that these partnerships are more structured and systematic, ensuring that apps meet the real needs of patients with SCI and their caregivers. Without this validation process, there is a risk of developing tools that do not effectively address the complexities of SCI self-management, which calls for a more standardized and research-driven approach to app development.

Furthermore, the study’s focus on English-language apps presents a potential limitation. While the authors acknowledge this constraint, restricting searches to English-language apps may exclude valuable resources tailored to non–English-speaking populations, limiting the generalizability of findings.

The integration of validation studies and collaborative research is fundamental to enhancing the effectiveness, usability, and real-world applicability of digital tools in SCI rehabilitation. Expanding this approach will ensure that these tools are not only evidence-based but also practical and adaptable to diverse patient needs.

The study identifies the absence of gamification elements in most reviewed apps, presenting an opportunity for innovation, as interactive learning approaches have demonstrated efficacy in enhancing engagement and knowledge retention [4].

I appreciate the authors’ valuable contribution to this evolving field and encourage future research to expand the scope of app evaluations to include caregiver-focused tools, multilingual resources, and usability considerations for individuals with functional impairments. These additions would provide a more comprehensive understanding of digital health solutions in SCI rehabilitation.

## Abbreviations

SCI: spinal cord injury
